# Alkaline water-splitting reactions over Pd/Co-MOF-derived carbon obtained *via* microwave-assisted synthesis

**DOI:** 10.1039/d0ra02307h

**Published:** 2020-05-05

**Authors:** Adewale K. Ipadeola, Kenneth I. Ozoemena

**Affiliations:** Molecular Sciences Institute, School of Chemistry, University of the Witwatersrand Private Bag 3, PO Wits Johannesburg 2050 South Africa Kenneth.ozoemena@wits.ac.za +27 11 717 6730

## Abstract

Cobalt-based metal–organic framework-derived carbon (MOFDC) has been studied as a new carbon-based support for a Pd catalyst for electrochemical water-splitting; *i.e.*, the hydrogen evolution reaction (HER) and oxygen evolution reaction (OER) in alkaline medium. The study shows a high increase in the HER activity, in terms of low onset overpotential (onset *η* = 35 mV *vs.* RHE), high exchange current density (*j*_o,s_ ≈ 0.22 mA cm^−2^), high mass activity (*j*_o,m_ ≈ 59 mA mg^−1^), high kinetic current (*j*_K_ ≈ 5–8 mA cm^−2^) and heterogeneous rate constant (*k*^0^ ≈ 4 × 10^−4^ cm s^−1^), which are attributed to the high porosity of MOFDC and contribution from residual Co, while the large Tafel slope (*b*_c_ = 261 mV dec^−1^) is ascribed to the high degree of hydrogen adsorption onto polycrystalline Pd as a supplementary reaction step to the suggested Volmer–Heyrovsky mechanism. These values for the catalyst are comparable to or better than many recent reports that adopted nano-carbon materials and/or use bi- or ternary Pd-based electrocatalysts for the HER. The improved HER activity of Pd/MOFDC is associated with the positive impact of MOFDC and residual Co on the Pd catalyst (*i.e.*, low activation energy, *E*_A_ ≈ 12 kJ mol^−1^) which allows for easy desorption of the H_ads_ to generate hydrogen. Moreover, Pd/MOFDC displays better OER activity than its analogue, with lower onset *η* (1.29 V *vs.* RHE) and *b*_a_ (≈78 mV dec^−1^), and higher current response (*ca.* 18 mA cm^−2^). Indeed, this study provides a new strategy of designing and synthesizing MOFDC with physico-chemical features for Pd-based electrocatalysts that will allow for efficient electrochemical water-splitting processes.

## Abstract 2

Palladium nanoparticles supported on MOF-derived carbon serve as an efficient bifunctional electrocatalyst for alkaline water-splitting reactions.

## Graphical abstract

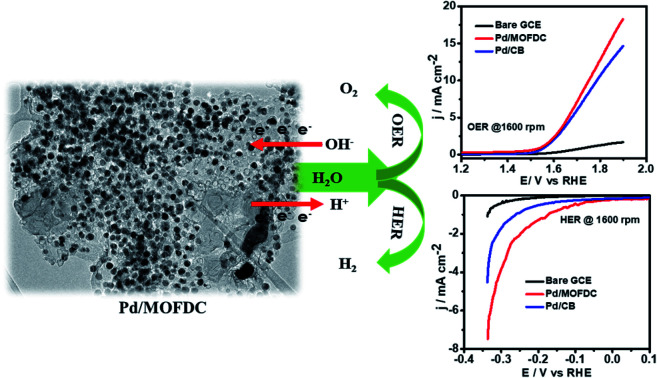

## 1. Introduction

Hydrogen is an excellent fuel for the realization of clean and renewable energy. Hydrogen evolution reaction (HER) *via* electrochemical water-splitting process has become important in a world that strives for a clean environment and urgent mitigation of the negative consequences of climate change brought about by greenhouse gases. Efficient HER is generally dependent on the nature of electrocatalysts that drive the reaction.

In alkaline media, the electrocatalyst requires energy to break the H–O–H bonding before chemisorption and desorption of hydrogen intermediate (H_ads_) to produce H_2_, as illustrated in eqn (1)–(3).^1^ The Volmer is the rate-determining step (RDS), which is initial H–O–H cleavage, followed by chemisorption of the H_ads_ onto active sites (M) of noble metal electrocatalyst, while the HER subsequently occur either by eqn (2) (Tafel) or (3) (Heyrovsky) depending on the nature of the electrocatalyst and values of Tafel slope for H_2_ production, as the H_ads_ recombine to yield H_2_ or the catalyst continuously cleave water molecule and desorbed the H_ads_ to produced H_2_, respectively. The latter (eqn (3)) may mostly be preferred for efficient HER electrocatalysis in alkaline conditions because of a limited amount of H_ads_ which could only be generated by splitting water.^2^1
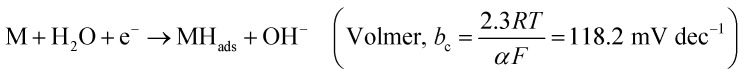
2
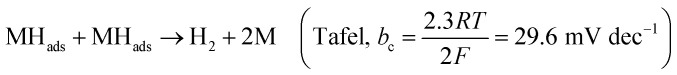
3
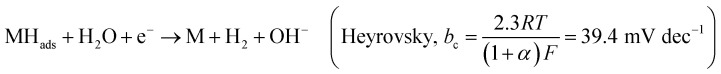


HER is extremely sluggish in alkaline compared to the acidic media due to the initial water dissociation, instability of adsorbed hydrogen (H_ads_) and ultimately large overpotential,^2^ hence explaining the preference of platinum (Pt)-based electrocatalysts for efficient catalysis of HER in alkaline electrolyte. According to density functional theory (DFT), the theoretical H_2_ binding energy (*E*_ads_) of palladium (Pd(111), *E*_ads_ = −0.48 eV) is close to that of Pt (Pt(111), *E*_ads_ = −0.43 eV), with excellent HER activities.^3^ Moreover, Pd-based electrocatalysts have proved to exhibit improved electrocatalytic performance in alkaline conditions.^4^ Hence, Pd-based electrocatalysts are viable substitute electrocatalysts for HER in an alkaline medium because of their high selectivity for H_2_ binding, rapid desorption of H_ads_, quick water splitting and transformable formation of H_ads_ to produce highly purified H_2_ gas.^5^

Presently, several attempts are being made to reduce the cost of HER by using low-Pd loaded electrocatalyst on various carbon supports including carbon fibre,^6^ carbon paper substrate,^7^ Vulcan XC-72,^8^ graphitic nanofiber,^9^ and reduced graphene oxide,^10^ to mention a few. Although reports on these carbon-based supports show good results, a lot still needs to be done to enhance the electrocatalytic performance in terms of current density, energetics and kinetics. To our knowledge, there are little reports on the use of metal–organic framework-derived carbons (MOFDC) for HER and OER in alkaline electrolyte.^11^ The choice of Co-MOF sacrificial template to synthesize novel carbon support (MOFDC) is because of the interesting inherent properties of MOF such as tunable pore size, enhanced surface area, electron density, electrical conductivity and thermal stability, and the ease of Co removal after carbonization by etching with acid.^12,13^

This work describes the first report on the effect of MOFDC as a support for Pd electrocatalyst for HER activities. It is clearly shown that MOFDC significantly enhances both the energetic and kinetics of HER when compared to its analogue and many literature reports using other carbon-based supports.

## 2. Experimental

**Fig. 1:**
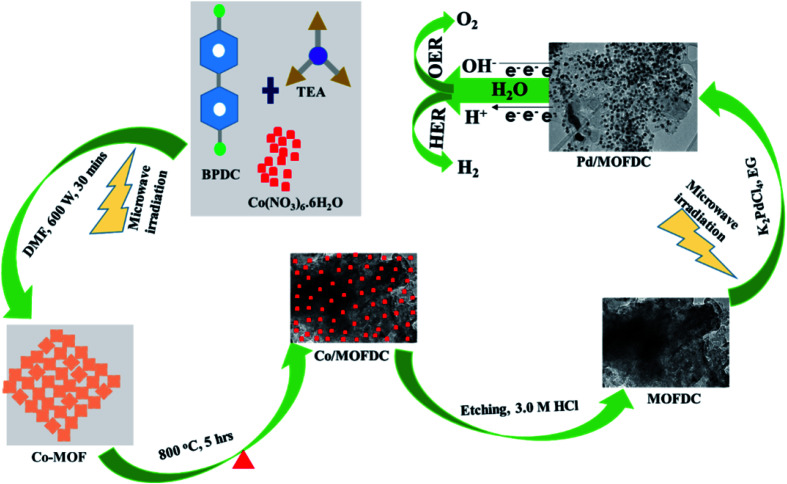
Schematic illustration for the synthesis of Pd/MOFDC and Pd/CB electrocatalysts.

Nanostructured palladium electrocatalyst supported on MOFDC (Pd/MOFDC) was synthesized using previously reported microwave-assisted synthesis (summarized in Fig. 1).^14^ In brief, cobalt nitrate hexahydrate (Co(NO_3_)_6_·6H_2_O, 0.4366 g), 4,4′-biphenyldicarboxylic acid (BPDC, 0.3634 g) and triethylamine (TEA, 1.5 mL) were mixed in a flask containing dimethylformamide (DMF, 50 mL) and stirred for 15 min to obtain a homogeneous mixture. The mixture was then heated in a microwave (600 W, 150 °C, 30 min) forming precipitates, which were washed and dried in vacuum (60 °C) to obtain solid cobalt-based metal–organic frameworks (Co-MOF). The Co-MOF was then annealed from room temperature to 600 °C at 5 °C min^−1^ and then to 800 °C and maintained for 5 h. The resulting solution formed was cobalt nanoparticles on metal–organic frameworks-derived carbon (Co/MOFDC). The Co/MOFDC was then chemically etched with hydrochloric acid (3.0 M, 10 mL) to remove the cobalt nanoparticles, resulting in MOFDC. Pd nanoparticles (20 wt%) were dispersed onto the MOFDC by following this procedure: potassium tetrachloropalladate crystals (K_2_PdCl_4_, 61.35 mg) were dissolved in EG (50 mL) and stirred for 30 min and pH of the solution was raised to 12 using NaOH (1.0 M). Then, the MOFDC (80 mg) was added and further stirred for 30 min. The mixture was irradiated with MW (600 W, 140 °C, 1 h) to disperse Pd nanoparticles on the support, after which the pH of the resulting product (Pd/MOFDC) was lowered to 3, using nitric acid (HNO_3_, 0.1 M). The final product was washed and dried (80 °C, 4 h) in a vacuum oven. A similar procedure was followed for making Pd/carbon black (CB).

The physical properties of the materials and electrocatalysts were thoroughly probed using Raman spectroscopy (Horiba LabRam HR Raman spectrometer equipped with Olympus BX41 microscope), powdered X-ray diffraction (Bruker D2 XRD), transmission electron microscope (FEI Tecnai T12 Sprint TEM), Brunauer–Emmett–Teller (BET, Micrometric Tristar 3000) and thermal gravimetric analysis (PerkinElmer TGA 4000). All electrochemical measurements were carried out using a rotating disk electrode (RDE) with AUTO LAB PGSTAT204. The HER kinetics on the Pd/MOFDC and Pd/CB were investigated utilizing a conventional three-electrode system: the working electrode is a glassy carbon electrode (GCE, 5 mm diameter) modified with the Pd/MOFDC (GCE-Pd/MOFDC) and Pd/CB (GCE-Pd/CB) while platinum wire and Ag|AgCl, 3.0 M KCl were employed as counter and reference electrodes, respectively. Before the measurements, electrocatalyst inks were prepared and modified on the GCE as follow: 2 mg of each electrocatalyst powder was dispersed in a mixture of ethanol (2 mL) and Nafion (5 wt%, 50 μL) and ultrasonically mixed for uniform distribution. Each electrocatalyst ink (20 μL) was coated on the polished surface of the GCE and allowed to dry in the air to afford Pd catalyst loading (0.0199 mg cm^−2^). The HER experiment was carried out in freshly prepared KOH electrolyte (0.1 M) at a controlled and varied temperature in water-bath thermostat. Moreover, electrochemical impedance spectroscopy (EIS) was used to confirm the HER kinetics at room temperature, 1600 rpm, overpotential 0.2 V *vs.* RHE and frequency range (100 kHz to 0.1 Hz). All potentials are referred to reversible hydrogen electrode (RHE) with conversion formula: RHE = *E*_Ag|AgCl_ + 0.197 + 0.059 pH, where pH = 12.57.

## 3. Results and discussion

### 3.1 Physical characterization: Raman, XRD, BET and TEM measurements

**Fig. 2:**
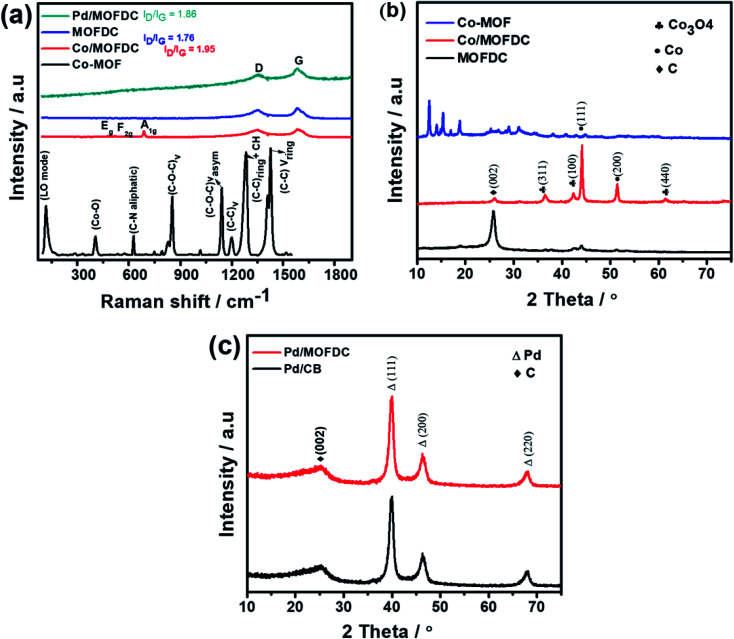
(a) Raman spectral of Co-MOF, Co/MOFDC, MOFDC, and Pd/MOFDC, XRD of (b) Co-MOF, Co/MOFDC and MOFDC, and (c) Pd/MOFDC and Pd/CB.

**Fig. 3:**
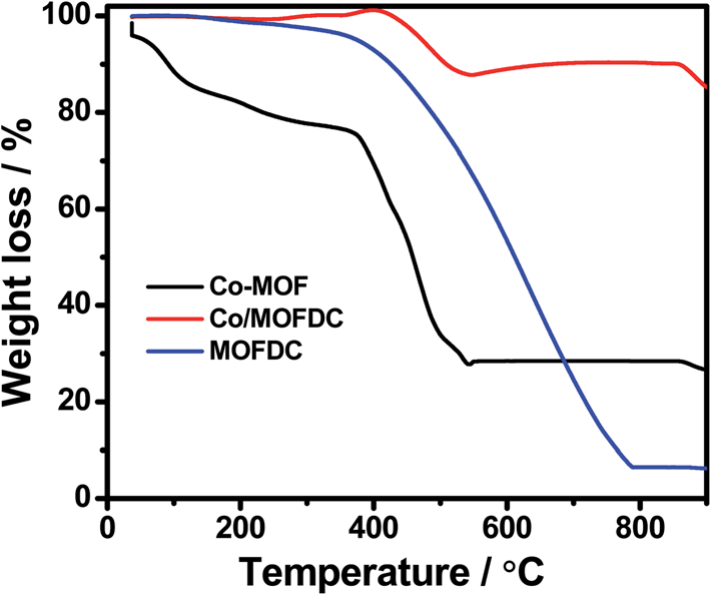
TGA profiles under air condition of Co-MOF, Co/MOFDC and MOFDC.

The chemical bond vibrations of the Co-MOF, Co/MOFDC, MOFDC and Pd/MOFDC were studied using Raman spectroscopy (Fig. 2a). The spectra show the Co–O linkage and the organic frameworks confirmed the synthesis of the Co-MOF.^14^ which was broken down after carbonization at high temperature, confirmed with TGA (Fig. 3), forming the Co/MOFDC with distinct vibrational modes of Co metal and D and G bands for the MOFDC. Chemically etched Co/MOFDC removed most of the Co nanoparticles, as only the D and G bands remain in the formation of MOFDC. Dispersion of Pd nanoparticles on the MOFDC shifted slightly the position of D and G bands and raised the *I*_D_/*I*_G_ value, showing increased defects.

Fig. 2b shows the XRD patterns of the Co-MOF, calcined product (Co/MOFDC) and derived carbon (MOFDC) obtained after chemical etching. The XRD pattern of the Co-MOF displays a well-crystalline material, which is fairly like the Co-MOF synthesized using benzene-1,3,5-tricarboxylic acid linkage.^15^ The slight difference in the crystallinity is ascribed to the difference in the architectural structure of chelating agents. After pyrolysis, the original peaks are replaced by series of peaks at 25.8° for hexagonal graphitic carbon (002), while the peaks at 36.5°, 42.6°, 44.0°, 51.7°, and 61.6° are attributed to a mixture of Co and Co_3_O_4_ nanoparticles with miller indices Co_3_O_4_ (311), Co_3_O_4_ (100), Co (111), Co (200) and Co_3_O_4_ (440) respectively.^16^ The chemically etched Co/MOFDC confirm that most Co and Co_3_O_4_ nanoparticles had been removed, giving rise to a pronounced carbon peak at 25.8° for the MOFDC (002) with little trace of peaks of Co/Co_3_O_4_ remaining. These results agree with the Raman spectral (Fig. 2a). The XRD patterns of the Pd/MOFDC and Pd/CB are described in Fig. 2c. As observed, the hexagonal carbon (002) at 25.6° remains for the Pd/MOFDC and Pd/CB. The electrocatalysts exhibit well-defined crystalline peaks for the face-centered cubic structure of Pd nanoparticles at around 40.0°, 46.5°, 68.0° for Pd(111), Pd(200) and Pd(220), respectively.^17,18^

Fig. 3 illustrates the TGA of the Co-MOF, Co/MOFDC and MOFDC. The TGA profile of the as-synthesized Co-MOF in air displays two decomposition steps. The first step indicates weight loss from room temperature to 134 °C, showing loss of water molecules from the pores of the Co-MOF because the Co-MOF has strong adsorption for water molecules.^16^ The second weight loss is observed from 372 °C to 541 °C, ascribable to the decomposition of the BPDC frameworks of the Co-MOF and formation of Co/MOFDC. These results are consistent with the previous report for Co-based MOF using 2,5-dihydroterephthalic acid (DHTP)^16^ and corroborate the Raman (Fig. 2a) and XRD (Fig. 2b) analysis for the decomposition of the Co-MOF frameworks at high temperature. The formation of Co nanoparticles in the second phase of the Co-MOF was confirmed from the TGA curve of the Co/MOFDC, where some of the metallic Co nanoparticles are oxidized between 400 °C and 541 °C to Co_3_O_4_ nanoparticles, as the analysis was done in the air, and Co residue (86.6 wt%) remains after 900 °C. The TGA profile of the MOFDC shows stability to 420 °C, where weight loss gradually begins to 800 °C and Co metal residue (6.5 wt%) remains after 900 °C. This observation is like the report obtained for carbon microsphere.^19^

**Fig. 4:**
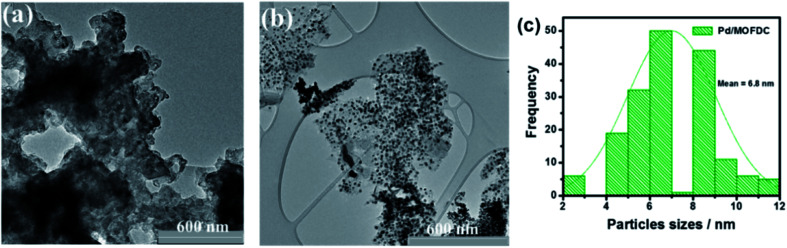
TEM micrographs of (a) MOFDC, (b) Pd/MOFDC and, particles sizes distribution (c) Pd nanoparticles.

Fig. 4 compares the TEM micrographs of the MOFDC and Pd/MOFDC. The morphology of the MOFDC with no traces of metal nanoparticles, as seen in Fig. 4a. Pd nanoparticles are revealed in Fig. 4b. The average particle size of Pd in the Pd/MOFDC is estimated to be 6.8 nm (Fig. 4c).

The specific surface area (*S*_BET_) of the Pd/MOFDC, MOFDC and Co-MOF are determined using BET analysis, summarized in Table 1. The Co-MOF has the lowest surface area and pore volume, but the highest pore size. After carbonization and chemically etched, the surface area and pore volume increased for the MOFDC, but the pore size reduced. Deposition of Pd nanoparticles on the MOFDC increased slightly the surface area and pore size while the pore volume decreased. The increased surface area and pore size in the Pd/MOFDC may be traced to microwave irradiation during its synthesis, while the decreased pore volume is expected as Pd nanoparticles fill some of the pores.^20^

**Table 1:** Comparative BET parameters for the precursor and electrocatalyst materials

BET analysis	Materials		
	Co-MOF	MOFDC	Pd/MOFDC
Specific surface area/m^2^ g^−1^	24.41	143.67	150.20
Pore volume/cm^3^ g^−1^	0.055	0.104	0.085
Pore size/nm	15.74	3.98	4.18

### 3.2 Electrochemical measurements

#### 3.2.1 Hydrogen evolution reaction (HER) study

**Fig. 5:**
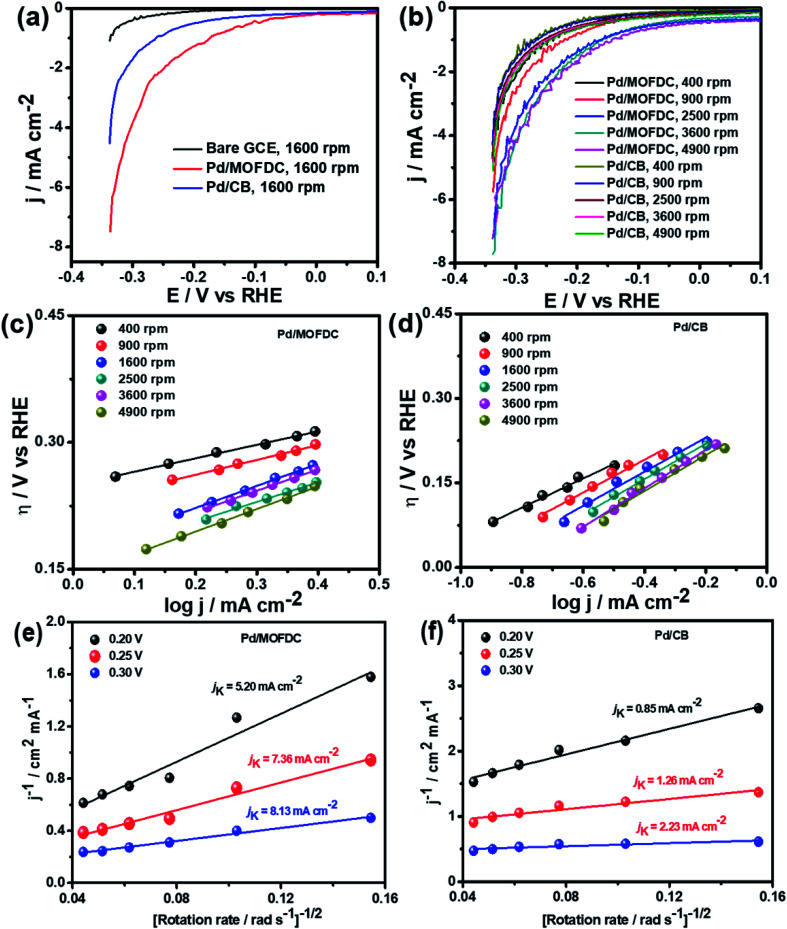
(a) HER polarization curves of bare GCE and the electrocatalysts at 1600 rpm, (b) the electrocatalysts at different rotations, 5 mV s^−1^ and 298 K, corresponding HER Tafel plots (c) Pd/MOFDC, (d) Pd/CB, and (e and f) Koutecky–Levich plots of the electrocatalysts in N_2_-deaerated 0.1 M KOH solution.

Electrochemical activities of the Pd/MOFDC and Pd/CB electrocatalysts toward HER were obtained using LSV at various rotation speeds (400, 900, 1600, 2500, 3600 and 4900 rpm) at a scan rate of 5 mV s^−1^ in N_2_-deaerated 0.1 M KOH electrolyte. Cathodic (HER) polarization curves of bare GCE and the electrocatalysts at 1600 rpm and other rotations for the electrocatalysts are illustrated in Fig. 5a and b, respectively. Current densities of the electrocatalysts increased for the HER in alkaline electrolyte, as the rotation speeds increased. The response of the Pd/MOFDC electrocatalyst is more than the Pd/CB at 1600 rpm (Fig. 5a) and all other rotations (Fig. 5b). These observations could be traced to the modification of electron properties of the Pd by the increased defect of the MOFDC (Fig. 2a) and contributions from the Co metal residue TGA (Fig. 3). The electrochemical active surface area (ECSA) of the Pd/MOFDC and Pd/CB electrocatalysts was determined by running CV in KOH solution only, then PdO reduction for the catalysts was integrated, evaluated and are previously recorded as 263.4 cm^2^ mg^−1^ and 235.3 cm^2^ mg^−1^, respectively.^14^

To understand the HER kinetics, the polarization curves were modelled to the typical Tafel equation, as given in eqn (4) and (5).4
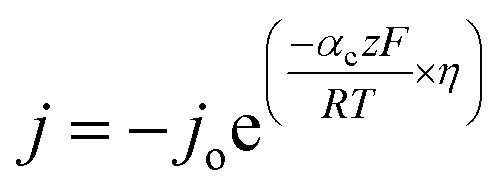
Taking the log of eqn (4), re-arranging and making the overpotential (*η*/V *vs.* RHE) as the subject lead to the cathodic and anodic Tafel equations (eqn (5) and (6), respectively):5
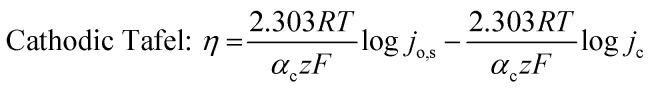
6
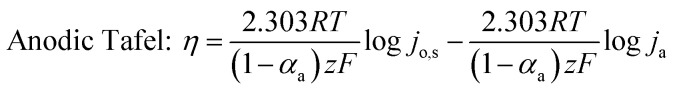
where *j*_c_ and *j*_a_ represent the cathodic and anodic current densities (mA cm^−2^), respectively; *j*_o,s_ is the exchange current density (mA cm^−2^), *R* is the universal gas constant, *T* is absolute temperature, *F* is Faraday constant, *α*_a_ and *α*_c_ are the anodic and cathodic electron transfer coefficients, *z* is the number of electrons involves in electrode reaction (*z* = 1). The Tafel plots of the electrocatalysts are presented in Fig. 5c and d. The kinetic parameters of the electrocatalysts for HER in N_2_-deaerated 0.1 M KOH are summarized in Table 2.

**Table 2:** Kinetic parameters of the electrocatalysts for HER in N_2_-deaerated 0.1 M KOH solution at different rotation, 298 K and 5 mV s^−1^

Kinetic parameters	Electrocatalysts	
	Pd/MOFDC	Pd/CB
**@400 rpm**		
*b*_c_/mV dec^−1^	159.3	258.0
*j*_o,s_/mA cm^−2^	0.1272	0.0614
*α*_c_	0.37	0.23

**@900 rpm**		
*b*_c_/mV dec^−1^	175.1	290.1
*j*_o,s_/mA cm^−2^	0.1506	0.0875
*α*_c_	0.34	0.20

**@1600 rpm**		
*b*_c_/mV dec^−1^	261.1	301.9
*j*_o,s_/mA cm^−2^	0.2226	0.1089
*α*_c_	0.23	0.20

**@2500 rpm**		
*b*_c_/mV dec^−1^	231.5	314.5
*j*_o,s_/mA cm^−2^	0.2030	0.1264
*α*_c_	0.26	0.19

**@3600 rpm**		
*b*_c_/mV dec^−1^	252.9	339.4
*j*_o,s_/mA cm^−2^	0.2185	0.1537
*α*_c_	0.23	0.17

**@4900 rpm**		
*b*_c_/mV dec^−1^	266.3	318.7
*j*_o,s_/mA cm^−2^	0.2943	0.1495
*α*_c_	0.22	0.19

The Pd/MOFDC electrocatalyst shows higher *j*_o,s_ values at all the rotations speeds than the Pd/CB electrocatalyst and compared literature (see Table 3) because of ease of continuous H–O–H cleavages and more facile desorption of H_ads_ from the active sites (M) of Pd and increased rates of H_2_ production, with agitation during HER as the rotation speed increased, as the M–H_ads_ bond is predominantly moderated by van der Waals forces.^21^ The H_ads_ present in this medium is continuously generated by electrocatalytically splitting water for H_2_ production. Compared to most reports, the Pd/MOFDC also shows higher mass activity (*j*_o,m_) of 58.63 mA mg^−1^ at 1600 rpm in alkaline condition, due to the ease of continuous water splitting, yielding MH_ads_, and subsequently desorption of the H_ads_ from the active sites of the Pd catalyst to eventually produce H_2_.^22^

**Table 3:** Comparison of electrochemical HER activity of the electrocatalysts with literature in the alkaline electrolytes at 1600 rpm and 5 mV s^−1^ in terms of onset overpotential (onset *η*), Tafel slope (*b*_c_), exchange current density (*j*_o,s_) and mass activity (*j*_o,m_)

Electrocatalysts	Electrolytes	Onset *η*/mV *vs.* RHE	*b*_c_/mV dec^−1^	*j*_o,s_/mA cm^−2^	*j*_o,m_/mA mg^−1^	Ref.
Pd/MOFDC	0.1 M KOH	35.2	261	0.2226	58.63	This work
Pd/CB	0.1 M KOH	56.7	302	0.1089	25.62	This work
Pd/C-500 °C	0.1 M KOH	60	148	0.1220	21.00	28
f-MWCNTs@Pd/TiO_2_	0.1 M KOH	<100	130	0.06	n/a	29
PdNiMo film	0.1 M KOH	85	227	n/a	n/a	22
rGO-Au_48_Pd_52_	0.1 M KOH	80	149	0.1300	50.00	30
Pd–CN_*x*_	0.5 M KOH	∼89	150	0.2450	16.00	31
Pd	1.0 M KOH	n/a	210	0.1259	n/a	32
NiCo_2_P_4_	1.0 M KOH	∼98	34.3	0.45	n/a	33
Pd–FeO_*x*_(OH_2_)_2−2*x*_	0.1 M KOH	50	135	0.17	n/a	34

Table 3 compares HER kinetics of the Pd/MOFDC with the Pd/CB and literature. The HER onset overpotential (onset *η*) values of 35 mV and 56.7 mV were obtained for the Pd/MOFDC and Pd/CB, with corresponding overpotential (*η*) values (at 2.5 mA cm^−2^) of 273.2 mV and 324.4 mV, respectively. The less *η* values of the Pd/MOFDC suggests lower energy is required for the evolution of hydrogen relative to other electrocatalysts, due to its ease of continuous water splitting for H_2_ production. Tafel slope of the electrocatalysts for the HER is a parameter employed to explain that the catalysts continuously split water, even after the initial water splitting (Volmer). The high Tafel slopes of the catalysts suggest that Heyrovsky reaction (eqn (3)) proceeds immediately after the RDS (eqn (1)).^23^ Thus, Volmer–Heyrovsky processes may predominate as the HER mechanism, as the process requires continuous H–O–H cleavage, adsorption of H (MH_ads_) and desorption of the H_ads_.^24^ This assumption agrees with the study of free energy diagram of HER in alkaline conditions, which proved that Volmer–Heyrovsky mechanism dominate.^25^ However, high Tafel slopes (160–266 mV dec^−1^) obtained here than the theoretical values for Volmer and Heyrovsky processes, have previously been ascribed to high hydrogen adsorption onto the active sites of polycrystalline Pd electrocatalysts, as supplementary reaction step,^26,27^ and are also reported in the compared literature (see Table 3).

Koutecky–Levich plots of the electrocatalysts are employed to separate kinetic current from the mass transfer as the rotation speed increased, shown in Fig. 5e and f at varied overpotentials 0.20, 0.25 and 0.30 V (*vs.* RHE). The kinetic currents (*j*_K_) were extrapolated from the intercept (1/*j*_K_), as it is potential-dependent. At the overpotentials studied, the Pd/MOFDC records *j*_K_ values of 5.20, 7.36 and 8.13 mA cm^−2^, while the Pd/CB records 0.85, 1.26 and 2.23 mA cm^−2^, respectively. Then, heterogeneous rate constants (*k*^0^) of the electrocatalysts were determined from the *j*_K_ values and averaged, following the relationship (*j*_K_ = *nFk*^0^*C*). Hence, mean *k*^0^ values of 3.57 × 10^−4^ and 7.53 × 10^−5^ cm s^−1^ were calculated for the Pd/MOFDC and Pd/CB, respectively. These results show relatively facile HER activity concerning high *j*_K_ and *k*^0^ values are also observed for the Pd/MOFDC, whereas the Pd/CB displays more sluggish HER kinetic. The same observation was recorded for the mass transfer with increased *j*_o,s_ and *j*_o,m_ values obtained for the Pd/MOFDC at all rotation speeds.

**Fig. 6:**
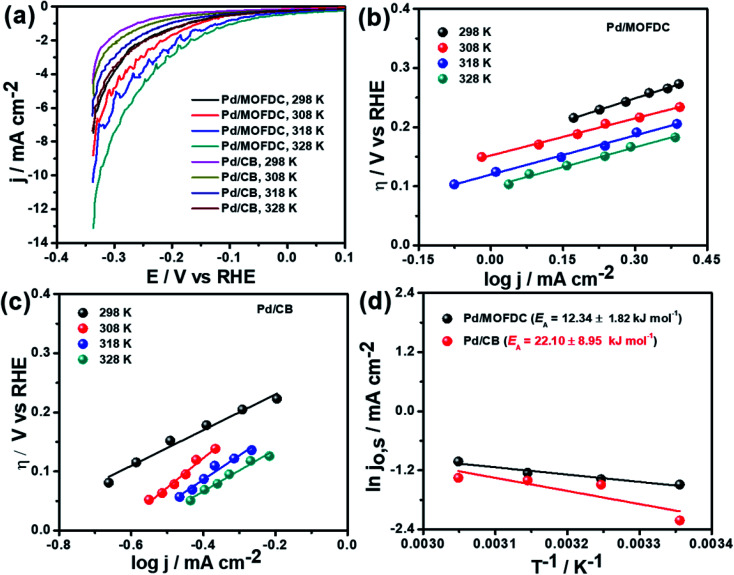
(a) HER polarization curves of the electrocatalysts at 1600 rpm, 5 mV s^−1^ and different temperatures, corresponding HER Tafel plots of (b) Pd/MOFDC, (c) Pd/CB and (d) Arrhenius plots of the electrocatalysts in N_2_-deaerated 0.1 M KOH solution.

Temperature-dependence HER activities on the electrocatalysts in N_2_-deaerated 0.1 M KOH solution at 1600 rpm and 5 mV s^−1^ were studied at four different temperatures (298, 308, 318 and 328 K). The HER polarization curves of the Pd/MOFDC and Pd/CB electrocatalysts are displayed in Fig. 6a. To understand the effect of temperature on the HER, the polarization curves were also modelled to the conventional Tafel plots (eqn (5)), as shown in Fig. 6b and c.

The kinetic parameters of the temperature-dependent HER in N_2_-deaerated 0.1 M KOH solution on the electrocatalysts, at 1600 rpm and 5 mV s^−1^, are summarized in Table 4. The *j*_o,s_ values for the HER increased with increasing temperature. At all the temperatures studied in this work, the Pd/MOFDC electrocatalyst showed extremely higher *j*_o,s_, relative to the Pd/CB and literature. This observation could be due to the incremental ease of continuous splitting of water on the Pd/MOFDC electrocatalyst as the temperature increases for H_2_ production. This hypothesis agrees with the previous report that H–O–H splitting is enhanced at elevated temperatures.^35,36^

**Table 4:** Temperature-dependence kinetic parameters of HER in N_2_-deaerated 0.1 M KOH solution on the electrocatalysts at 1600 rpm, 5 mV s^−1^ and different temperatures

Kinetic parameters	Electrocatalysts	
	Pd/MOFDC	Pd/CB
**@298 K**		
*b*_c_/mV dec^−1^	261.1	301.9
*j*_o,s_/mA cm^−2^	0.2226	0.1089
*α*_c_	0.23	0.20

**@308 K**		
*b*_c_/mV dec^−1^	205.1	496.5
*j*_o,s_/mA cm^−2^	0.2513	0.2255
*α*_c_	0.30	0.12

**@318 K**		
*b*_c_/mV dec^−1^	220.9	409.8
*j*_o,s_/mA cm^−2^	0.2871	0.2455
*α*_c_	0.29	0.15

**@328 K**		
*b*_c_/mV dec^−1^	222.9	353.5
*j*_o,s_/mA cm^−2^	0.3597	0.2585
*α*_c_	0.29	0.27

Arrhenius equation was further employed to determine the minimum amount of energy required (activation energy (*E*_A_)) for the electrocatalysis of HER to occur. Fig. 6d indicates the Arrhenius plots (plot of the natural logarithm of exchange current (ln *j*_o,s_) against the reciprocal of temperature (*T*^−1^)) where the *E*_A_ was extrapolated from the slope 
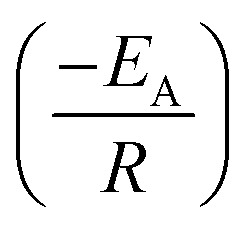
 and calculated as 12.34 ± 1.82 kJ mol^−1^ and 22.10 ± 8.95 kJ mol^−1^ for the Pd/MOFDC and Pd/CB, respectively. The values of the *E*_A_ for these Pd-based catalysts were less than those previously reported for Pt/C (≈18 kJ mol^−1^).^37^ The small value of the *E*_A_ for the Pd/MOFDC indicates reduced energy is required for continuous water splitting, perhaps due to lowered hydride phase formation for rapid production of H_2_,^37^ which is consistent with its onset overpotential and overpotential at 2.5 mA cm^−2^. These observations are expected as Density Functional Theory (DFT) previously proved that the binding energy of hydrogen onto Pd-based catalyst is lowered with carbon supports that have increased electrons density.^38,39^

**Fig. 7:**
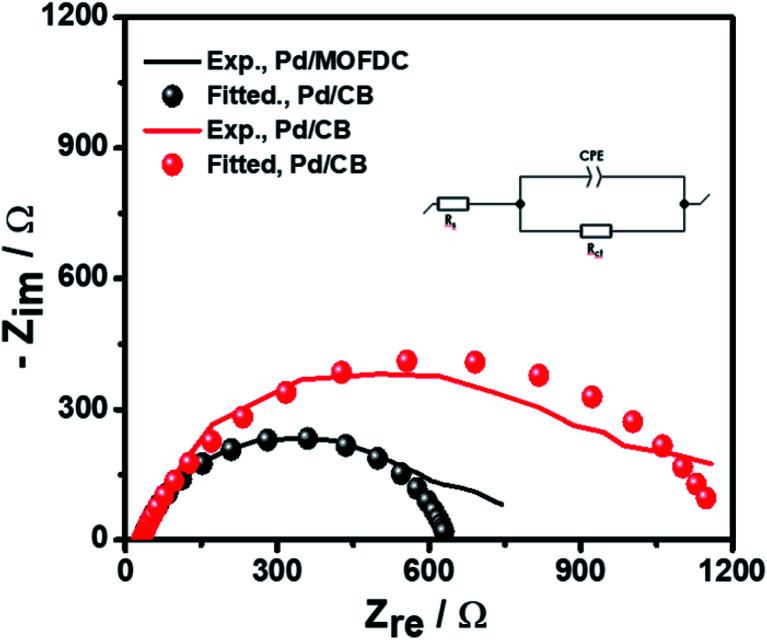
EIS studies of the electrocatalysts for HER at overpotential 0.2 V, 1600 rpm, 5 mV s^−1^ and 298 K.

Further investigation of the electrocatalysts for HER kinetics at room temperature, 1600 rpm and overpotential of 0.2 V *vs.* RHE was done using the EIS studies. The Nyquist plots (Fig. 7) with the modelled electrochemical equivalent circuit (inset) employed for fitting, revealed the HER kinetic of the electrocatalysts (listed in Table 5) during this process. The most important parameter is the charge transfer resistance (*R*_ct_), which is used to determine the *j*_o,s_, following eqn (7) below.7
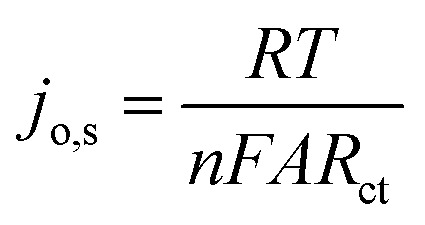


**Table 5:** EIS kinetic data of the electrocatalysts for HER at overpotential 0.2 V *vs.* RHE, 1600 rpm, 5 mV s^−1^ and 298 K

EIS parameters	Electrocatalysts	
	Pd/MOFDC	Pd/CB
*R*_s_/Ω	32.64 ± 0.27	31.30 ± 0.26
*R*_ct_/Ω	603.20 ± 0.59	1160.00 ± 032
CPE/μF s^(1−*a*)^	79.93 ± 0.83	117.00 ± 0.49
*a*	0.84	0.79
*χ* ^2^/|*Z*|	1.68	1.66
*j*_o,s_/mA cm^−2^	0.2172	0.1129

The *R*_ct_ values of 603.20 Ω and 1160.00 Ω, resulted in calculated *j*_o,s_ values of 0.2172 and 0.1129 mA cm^−2^ for the Pd/MOFDC and Pd/CB, respectively. These values are approximately close to the kinetic values obtained using the Tafel plots.

#### 3.2.2 Oxygen evolution reaction (OER) study

**Fig. 8:**
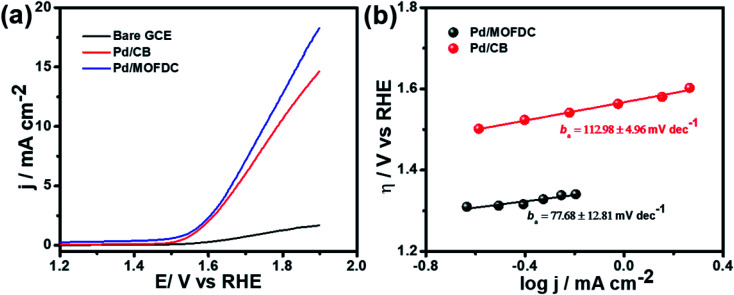
(a) OER polarization curves and (b) OER Tafel plots of the electrocatalysts in N_2_-deaerated 0.1 M KOH at 1600 rpm, 5 mV s^−1^ and 298 K.

Out of curiosity, we also checked the ability of the Pd/MOFDC and Pd/CB to perform the oxygen evolution reaction (OER). As shown in Fig. 8a, the OER polarization curves for the electrocatalysts were conducted at 1600 rpm, 5 mV s^−1^ and room temperature. This polarization curve was modelled to Tafel plots (eqn (6)), as shown in Fig. 8b. The data show a satisfactory current densities (*j*) of 18.40 mA cm^−2^ and 14.68 mA cm^−2^ with onset *η* of *ca.* 1.29 V and 1.49 V *vs.* RHE, which are higher than thermoneutral potential of water (1.23 V *vs.* RHE) for easy water splitting,^40,41^ and lowered than previously reported onset *η* value for the OER in the same medium,^42^ while Tafel slopes (*b*_a_) of 77.68 ± 12.81 mV dec^−1^ and 112.30 ± 4.96 mV dec^−1^ for the Pd/MOFDC and Pd/CB, respectively, as the large differences of these values, could be traced to the Co metal residue. The low Tafel slope of the Pd/MOFDC is associated with better OER activity than Pd/CB.^41,43^ These results show that the Pd/MOFDC electrocatalyst is not only good for HER but also excellent for OER, explaining its bi-functional behaviour for water electrolysis.

## 4. Conclusions

This work describes the first study of the use of MOFDC as efficient support for Pd electrocatalyst for HER and OER. The study reveals that the HER and OER activities on the Pd/MOFDC electrocatalyst are greatly improved, with extremely higher current densities compared to Pd/CB and literature. The enhanced HER activity of the Pd/MOFDC is attributed to the electronic properties of the Pd being modified by the increased electron density of the MOFDC and Co metal residue, resulting in the ease of continuously splitting of water to yield MH_ads_ and subsequently desorption of the H_ads_ to produce H_2_, evidenced from the increased mass transfer (*j*_o,s_ and *j*_o,m_) and kinetic (*j*_K_ and *k*^0^) parameters and the energy required to achieve this is very low. This observation was corroborated with the less *E*_A_ value of the Pd/MOFDC (12.34 ± 1.82 kJ mol^−1^) than the Pd/CB (22.10 ± 8.95 kJ mol^−1^). Additionally, the Pd/MOFDC displays satisfactory OER activity than its analogue, with lower onset *η* and *b*_a_, and higher current response. This new study provides an opportunity for further research to rationally design more MOFDCs using different ligands and transition metals for the efficient electrochemical water-splitting process.

## Conflicts of interest

There are no conflicts to declare.

## Supplementary Material
